# An Analysis of Trainees’ Operative Experiences Over the Past 16 Years Based on the Requirements of the New 2021 Cardiothoracic Surgery Curriculum in the United Kingdom and Ireland

**DOI:** 10.1093/ejcts/ezaf228

**Published:** 2025-07-09

**Authors:** Jeremy Chan, Daniel P Fudulu, Tim Dong, Hunaid A Vohra, Gianni D Angelini

**Affiliations:** Bristol Heart Institute, University of Bristol, Bristol BS2 8ED, United Kingdom; Bristol Heart Institute, University of Bristol, Bristol BS2 8ED, United Kingdom; Bristol Heart Institute, University of Bristol, Bristol BS2 8ED, United Kingdom; Bristol Heart Institute, University of Bristol, Bristol BS2 8ED, United Kingdom; Bristol Heart Institute, University of Bristol, Bristol BS2 8ED, United Kingdom

**Keywords:** operative experience, surgical education, United Kingdom

## Abstract

**Objective:**

Before introducing the new 7-year curriculum in 2021, cardiothoracic surgery trainees were required to complete a 2-year basic surgical training programme followed by 6 years of higher speciality training. The new curriculum eliminates the need for basic surgical training. It is outcome-based, requiring a minimum of 250 major cases performed as the first operator. We assess trainees’ operative experiences over the past 16 years based on the requirements of the new curriculum. The impact of the COVID-19 pandemic on training was also investigated.

**Participants:**

All trainees who commenced higher cardiothoracic surgical training from 2007 and graduated were included. Operative volumes were categorised into 13 major procedures (6 cardiac and 7 thoracic). The total number of major cases logged and the time required to achieve 250 cases were evaluated.

**Results:**

A total of 290 trainees were included, of whom 145 (50%) had completed training with validated eLogbooks. The median number of cases logged was 378 (interquartile range [IQR]: 309, 474) for all trainees across their training period. Cardiac-themed trainees logged 378 (IQR: 312.5, 474.5) cases, of which 202 (IQR: 166.5, 257.0) were coronary artery bypass grafting, followed by aortic valve surgery (*n* = 39, 14%, IQR: 15, 54.5). Thoracic-themed trainees logged 383.5 (IQR: 304.5, 469.75) cases, of which 345 (IQR: 270.25, 457.75) were anatomical lung resections followed by surgery for secondary pneumothorax (*n* = 124, 35%, IQR: 100.25, 165.0). The median time required to achieve 250 cases was 6.25 and 5.25 years for cardiac and thoracic trainees, respectively. There was an increase in time for trainees to complete higher surgical training before and during/after the COVID-19 pandemic (median 6 [IQR: 5, 8] vs 7 [IQR: 6, 10] years, *P* = 0.01).

**Conclusion:**

The new 2021 curriculum’s target of 250 major cases appears feasible. However, post-COVID-19 reductions in surgical volume and the removal of basic surgical training require integration and reform to ensure trainees can complete the programme in the 7-year timeframe.

## Graphical abstract

**Figure:**
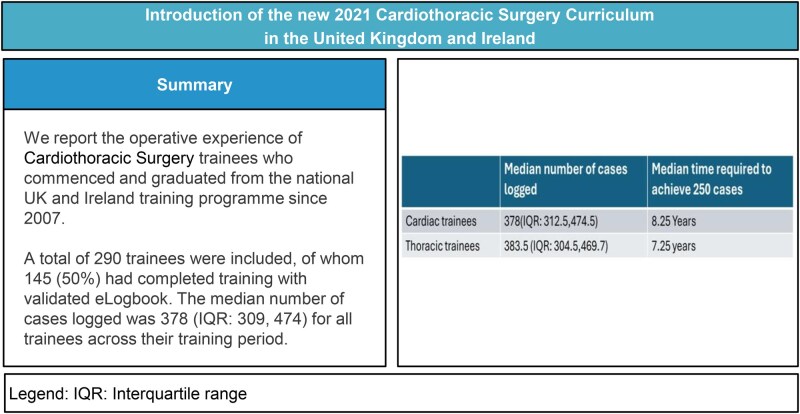

## INTRODUCTION

The United Kingdom and Ireland cardiothoracic surgery (CTS) curriculum underwent a significant reform in August 2021, following approval by the General Medical Council (GMC) and the Medical Council of Ireland (MCI).^1^ The curriculum transitioned from a time-based to an outcome-based model, reducing the expected training period from 8 to 7 years. To meet the revised curriculum requirements, trainees must select cardiac or thoracic surgery as their special interest by their fourth year of speciality training (ST4). The cardiothoracic training and the curriculum in the United Kingdom and Ireland are further explained in the discussion.

Additionally, a requirement set in the new curriculum is that trainees complete at least 250 major surgical cases as first operator, predominantly within their chosen speciality, to ensure surgical competency within the 7-year timeframe.^1^ The previous curriculum did not recommend a specific number of procedures. However, there are concerns about whether this condensed timeframe is sufficient for achieving these standards. The recruitment has also shifted from trainees who completed a 2-year basic surgical training to those who have just completed foundation training (equivalent to internship in the United States/Europe).

The COVID-19 pandemic caused unprecedented disruption to healthcare systems globally, and its impact on surgical training was profound.^2^ Although the new curriculum was finalized in November 2020, its implementation was postponed until 2021 due to the pandemic. The pandemic reportedly resulted in a substantial reduction in job satisfaction and clinical experience, as reflected in a national annual survey by CTS trainees,^3^ raising questions about how the pandemic may have influenced their ability to meet training requirements post-pandemic.

This study aimed to:

1. Assess trainees’ operative experiences over the past 16 years based on the new 2021 CTS curriculum requirements in the United Kingdom and Ireland.
2. Examine the impact of the COVID-19 pandemic on cardiothoracic surgical training.

## METHODS

This study utilized data from 3 UK-based national surgical training databases: the Joint Committee on Surgical Training (JCST), the Surgeons Information Management System (SIMS), the Intercollegiate Surgical Curriculum Programme (ISCP), and the Intercollegiate Surgical eLogbook. These datasets were linked using trainees’ GMC numbers and anonymized before analysis.

### Data sources

The ISCP Database (https://www.iscp.ac.uk/default.aspx)

Introduced in 2007, the ISCP functions as an online portfolio where trainees document work-based assessments, audits, teaching evaluations, and placement details. It also includes supervisor feedback and the Annual Review of Competence Progression (ARCP) outcomes. A summary of the ARCP outcomes is provided in **Supplementary Table S2**.^4^

The eLogbook Database: (https://www.elogbook.org/)

Operated by the Royal College of Surgeons of Edinburgh, the eLogbook allows trainees and consultants to record surgical procedures electronically, including supervision codes (eg, “Performed,” “Supervised-Trainer Scrubbed”). The database integrates with ISCP, enabling real-time tracking of trainee progress. Trainees can choose to opt out of sharing the data for research purposes.

The JCST SIMS Database: (https://www.jcst.org/)

This database stores demographic and training details for all surgical trainees, including start and predicted completion dates, deanery (Region of training, ie, London, South West) affiliations, and ARCP outcomes.

### Study population

All CTS trainees registered for speciality training from August 2007 to 2023 in the United Kingdom and Ireland were included in this study. The start date of year 1 of speciality training in CTS (ST1) or ST3/4 training was defined from data in both the JCST SIMS (registered start of speciality training date) and ISCP (start of placement date) databases. Completion of the training programme was defined as trainees obtaining an ARCP outcome 6 (Gained all the required competencies for completion of training).

### Study outcomes

The primary outcome for this study was to assess the overall cardiac- and thoracic-themed trainees’ operating experience based on the new 2021 United Kingdom and Ireland CTS curriculum.

The volume of cases logged in the eLogbook by trainees was examined. The cases logged were summarized into 13 major cases as listed in the 2021 curriculum (6 cardiac, 7 thoracic); details of the major cases are listed in **Table 1**, and full details are provided in **Supplementary Table S1**. The cases were included for analysis with the following supervision codes: S-TS (Supervised-trainer scrubbed), S-TU (Supervised-trainer not scrubbed), P (Performed), and T (Training more junior trainee). The total number of major cases performed as first operator, the time required to obtain ARCP outcome 6, and the award of Completion of Certificate of Training (CCT) were evaluated. Procedures performed before trainees entered the training programme were excluded based on the trainees’ start date. Trainees were further separated based on their subspeciality of choice (cardiac/thoracic surgery themed). Sub-analysis was performed comparing cardiac- and thoracic-themed trainees during the 2 time periods listed above.

**Table 1.** The Summary of Major Cases Listed in the 2021 Cardiothoracic Surgery Curriculum

Cardiac surgery major cases
● Isolated or concomitant coronary artery bypass grafting (CABG) ● Isolated or concomitant valve repair or replacement ● Thoracic aortic surgery ● Other major cardiac surgical cases involving cardiopulmonary bypass (CPB) ● Implantation/retrieval of the heart or lung (transplantation) ● Any congenital cardiac procedure

Thoracic surgery major cases
● Anatomical lung resection (open, video-assisted thoracoscopic surgery [VATS] or robotic) ● Correction of pectus deformity ● Decortication ● Thoracotomy for trauma ● Chest wall resection and reconstruction ● Tracheal resection ● Surgery of secondary pneumothorax (VATS/open)

The time in training for trainees was also reviewed to identify trainees who completed all training before the pandemic or part of the training period during the pandemic. The pandemic period was defined between March 2020 and March 2022, when all restrictions on all 4 nations in the United Kingdom and Ireland were lifted. The ARCP outcomes 10 were implemented explicitly due to the disruption to training caused by COVID-19. Outcome 10.1 was recommended for trainees who have progressed in their training but have been delayed in acquiring competencies/capabilities due to COVID-19. Outcome 10.2 was awarded to trainees at a critical progression point, and it would either be unsafe or impossible for the trainee to progress in or complete their training programme. Therefore, additional training time was required.^4^ It is worth mentioning that COVID-19 outcomes will not be rewarded in 2024.

Lastly, the median time of higher surgical training (speciality training year 3+) was plotted against the number of major cases logged by cardiac- and thoracic-themed trainees to examine the time required in training to fulfil the 250 major cases as the first operator recommended by the 2021 curriculum.

### Ethical statement

The JCST Data Analysis, audit and research group approves this project. The application complies with the UK General Data Protection Regulation. The application process, guidance, and previously approved projects are available on the JCST website. The study was performed following the ethical standards as laid down in the 1964 Declaration of Helsinki and its later amendments. The General Data Protection Regulations were strictly followed for the use of all data. There is no patient involvement in this study, and hence, patient consent is not required.

### Statistical analysis

Continuous variables are reported as a combination of mean and standard deviation (SD), as well as median and interquartile range (IQR). Categorical variables are reported as frequencies and percentages. Wilcoxon rank-sum test was used for comparison between 2 means of continuous, independent samples.

R (Version 4.1.1) and R Studio (Version 1.4.1103, RStudio, PBC) were used to perform the statistical analysis. Graphs and tables were created using R Studio (Version 1.4.1103, RStudio, PBC) and Microsoft Office 365 (Version 16.0.14026, 64 bits).

## RESULTS

### General characteristics

A total of 290 trainees were included in this study, of which 214 (73.79%), 75 (25.86%), and 1 (0.34%) were male, female, and prefer not to disclose, respectively. Ninety-three (31.03%) trainees entered the training at the ST1 level, while the rest (68.97%) began the training at the ST3/4 level. Trainees from all CTS training deaneries (England, Wales, Scotland, Northern Ireland) were included.

One hundred sixty-seven trainees (57.59%) had completed the training, with a median time of 7 (IQR: 5, 8) years of higher surgical training (from ST3) required to achieve all the competencies required to complete the training. The median time for cardiac- and thoracic-themed trainees to complete the higher surgical training was 7 (IQR: 6, 8) and 6 (IQR 5, 7) years, respectively. Of those, 29 trainees (17.58%) have taken time out of the training programme during their training period.

### Logbook experience at completion

Of the 167 trainees who completed the training, logbook details were available for 145 trainees (86.82%). Ninety-nine were cardiac-themed, and 46 were thoracic-themed.

The median number of major cases performed as the first operator was 378 (IQR: 309, 474) for all trainees across their training period. Cardiac-themed trainees performed a total of 378 (IQR: 312.5, 474.5) cases during their training period, of which 314 (IQR: 240.5, 379.5) were cardiac major cases. Thoracic-themed trainees logged a total of 383.5 (IQR: 304.5, 469.75) cases during their training period, of which 345 (IQR: 270.25, 457.75) were thoracic major cases.

**Figure 1.**
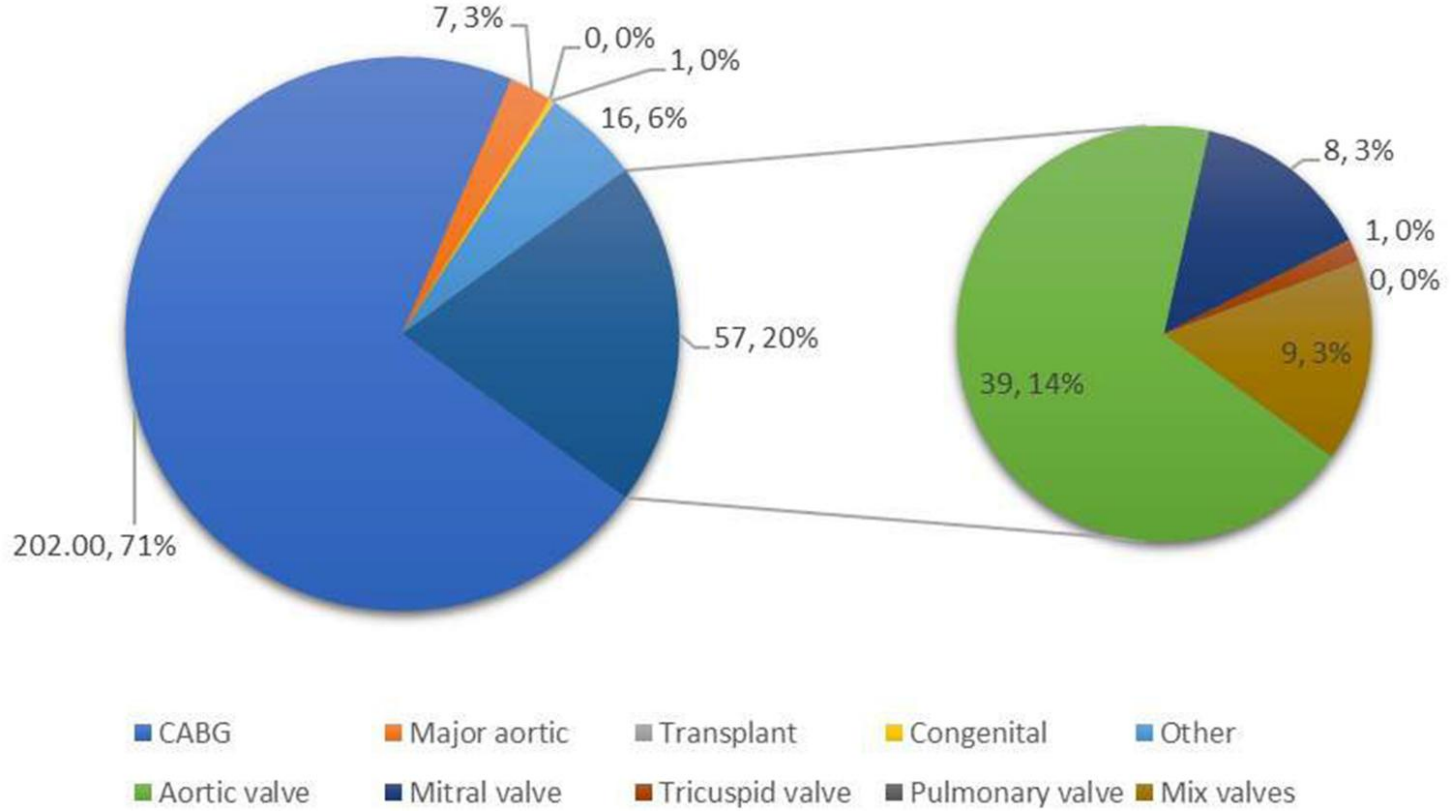
The Proportion of Cases Logged by Cardiac-themed Trainees Who Completed the Training Programme Based on the Examples of Major Cases Listed in the New 2021 Cardiothoracic Surgery Curriculum

**Figure 2.**
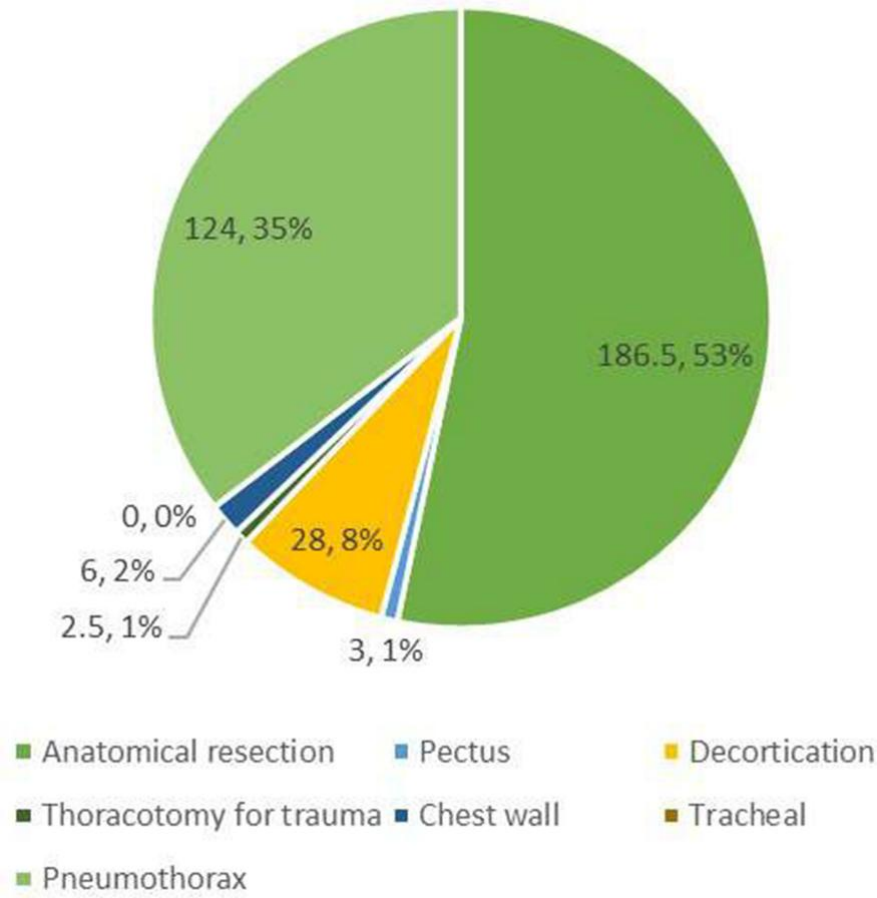
The Proportion of Cases Logged by Thoracic-themed Trainees Who Completed the Training Programme Based on the Examples of Major Cases Listed in the New 2021 Cardiothoracic Surgery Curriculum

The proportion of major cases performed as first operator by cardiac- and thoracic-themed trainees during their training period is shown in **Figures 1 and 2**. The most common major cases logged by cardiac-themed trainees were coronary artery bypass grafting (CABG; 202 cases, 71%), followed by aortic valve replacement/repair (AVR; 39 cases, 14%) and other major cardiac surgical procedures (requiring the use of cardiopulmonary bypass) (16 cases, 6%). Thoracic-themed trainees predominantly performed anatomical lung resection (186.5 cases, 53%), followed by surgery for secondary pneumothorax (124 cases, 35%) and decortication (28 cases, 8%).

Examining the logbook at completion, trainees registered to be first assistants in 52% of cases performed by their trainers. They performed 31% of cases under supervision and 17% without direct supervision, mainly in the last 2 years of training.

### Training progression and duration required for completion of training

**Figure 3.**
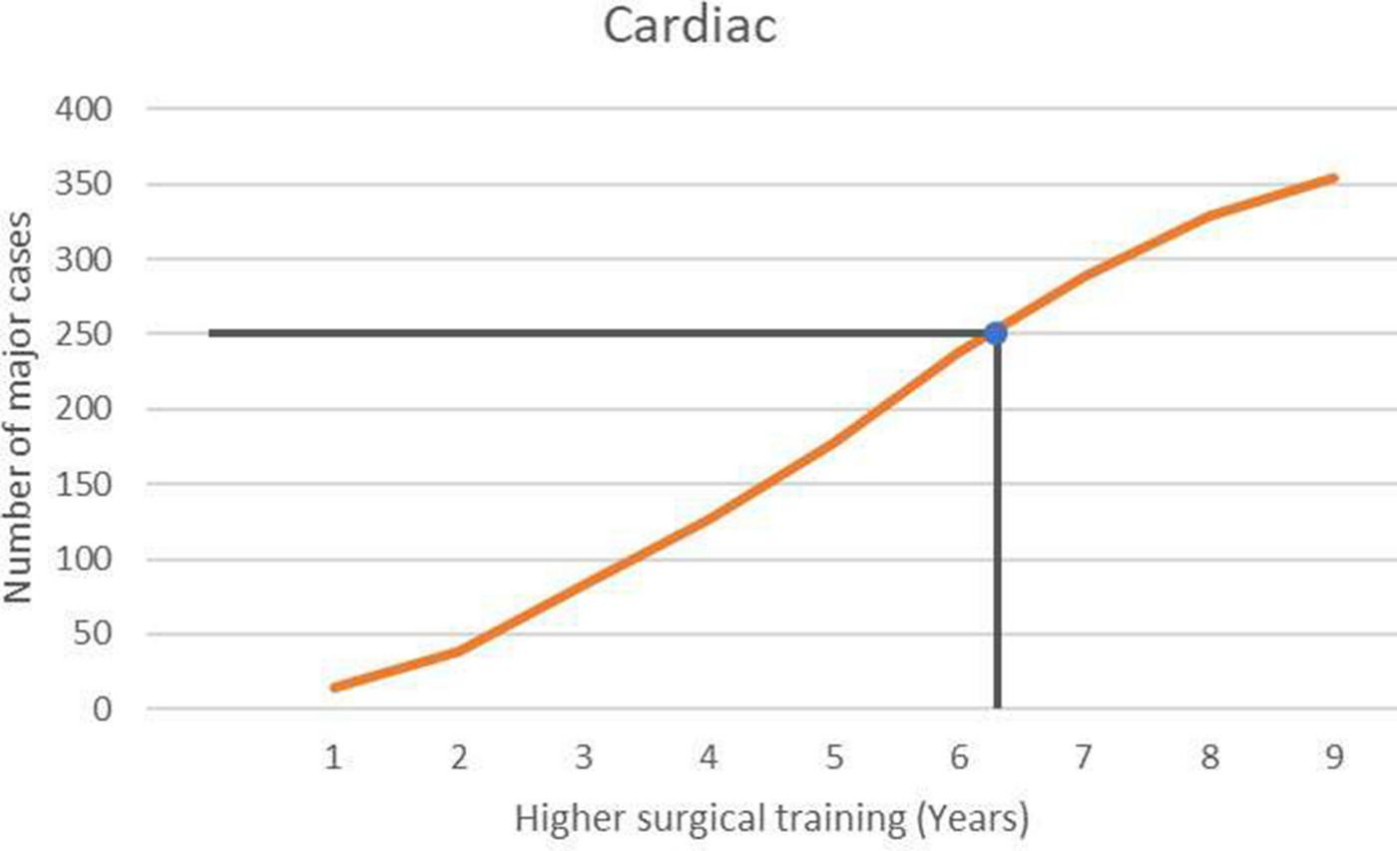
The Median Number of Major Cases Logged Against the Year of Higher Surgical Training for Cardiac-themed Trainees

**Figure 4.**
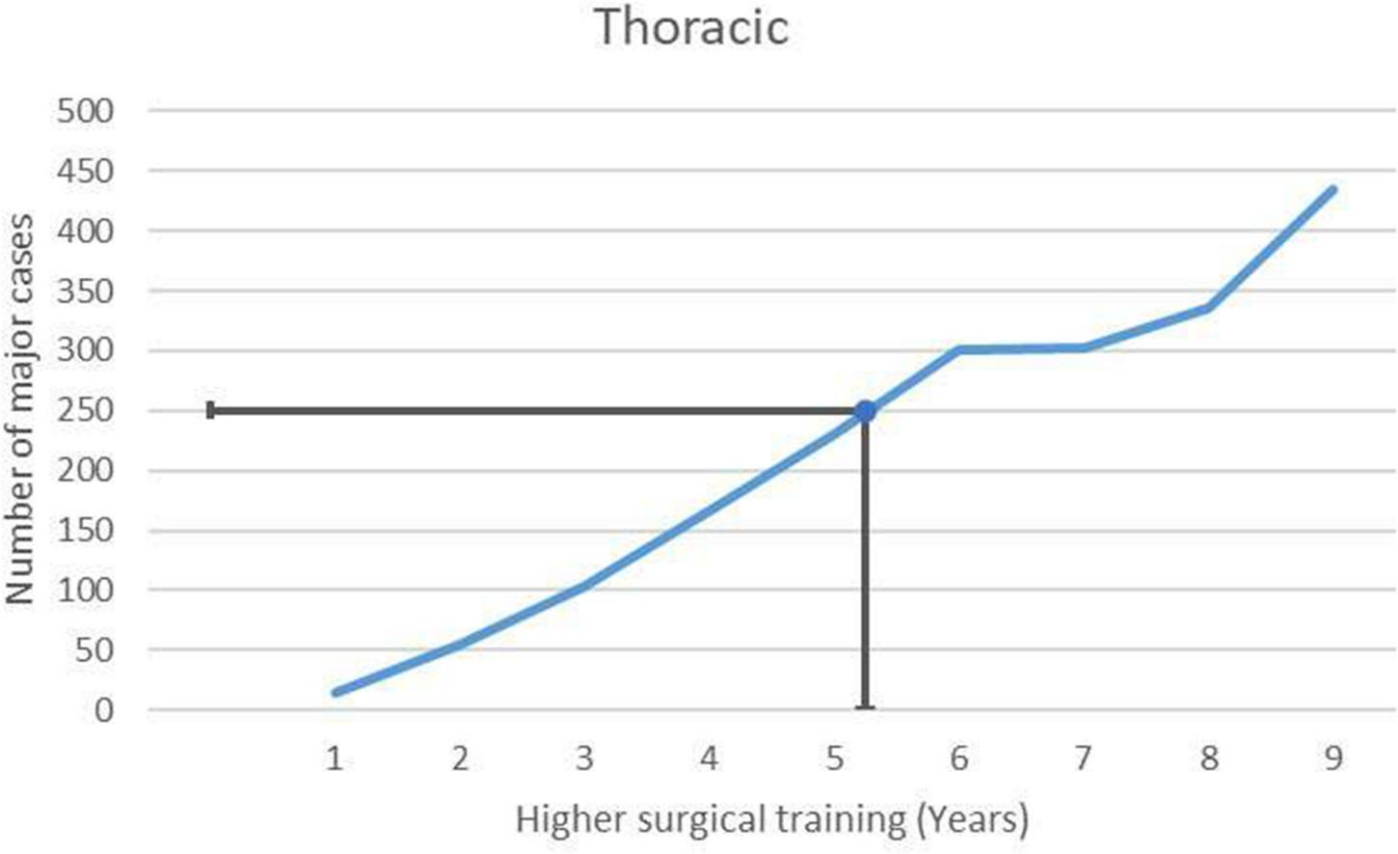
The Median Number of Major Cases Logged Against the Year of Higher Surgical Training for Thoracic-themed Trainees

The median time required for cardiac- and thoracic-themed trainees to undertake 250 major cases as first operators was 6.25 and 5.25 years of higher surgical training, respectively (**Figures 3 and 4**).

### Impact of COVID-19 on trainees’ completion of the training programme

Of the 290 trainees included, 177 (61.03%) had part of their training period during the pandemic. Seventy-nine trainees (44.6%) received a COVID ARCP outcome(s). A total of 54 and 40 ARCP 10.1 (trainees who have progressed but were delayed due to COVID-19) and 10.2 outcomes (trainees at critical progression point, unable to progress or complete the training programme) were awarded from 2020 to 2023, respectively.

Specifically, looking at the 167 trainees who obtained an ARCP outcome 6, 99 (59.28%) and 68 (40.72%) trainees completed the training before and during/after the COVID-19 pandemic, respectively. There was an increase in time for trainees to complete higher surgical training before and during/after the COVID-19 pandemic (median 6 [IQR: 5, 8] vs 7 [IQR: 6, 10] years, *P* = 0.01).

## DISCUSSION

Our study demonstrated that trainees who completed their cardiothoracic surgical training in the United Kingdom and Ireland had good exposure to the volume of major surgical procedures. The time required to achieve the new standard of 250 major cardiothoracic cases was approximately 6-7 years of higher surgical training (excluding the 2 years of basic surgical training). This raises the question of whether trainees can complete the programme within the intended 7 years of the new curriculum.

### CTS training in the United Kingdom and Ireland

CTS training in the United Kingdom and Ireland remains a popular and competitive programme and attracts many local and international applicants annually.^5^ In 2024 entry, 408 applications were received by the national recruiter (Health Education England) for 9 posts in the whole United Kingdom and Ireland, making CTS one of the most competitive surgical specialities.^5^ Successful applicants are placed into 1 of the 14 deaneries/regions and will work within the cardiothoracic centres within the deanery, with options to be trained outside the deanery. Each deanery has a training programme director who oversees the trainees’ progression and ensures that trainees meet the curriculum’s requirements. The recruitment has also shifted from recruiting trainees who completed a 2-year basic surgical training (year 4 post-graduation) to trainees who have just completed foundation training (equivalent to internship in the United States/Europe).

The full details of CTS training were previously described.^6^ Before the introduction of the new 7-year curriculum, trainees were required to complete a 2-year basic surgical training followed by a 6-year higher surgical training in CTS (similar to the 4 years of general surgery + 3 years of CTS in the United States).^6^ A run-through 8-year programme was first introduced in 2013 (similar to the integrated 6-year training in the United States). Regardless of the programme entry, the first 2 years aim for trainees to achieve basic surgical training and pass the membership of the Royal College of Surgeons examination.

In both the old and the newly introduced curriculum, all trainees’ progress is assess in the ARCP. This includes (but is not limited to) the operative experience logged in the eLogbook database and the number of work-based assessments completed in the ISCP database. In addition, extra-curricular activities such as teaching, publication, national presentations, and audits are considered during the ARCP assessment. By the end of the training, if trainees have met all the requirements, they will be rewarded with an ARCP outcome 6 and certification in CTS. Some requirements include clinical experience, operative experience (250 index procedures), passing the fellowship examination, and knowledge in critical conditions. Full details are included on page 34 of the CTS curriculum.^1^

### CTS curriculum

**Figure 5.**
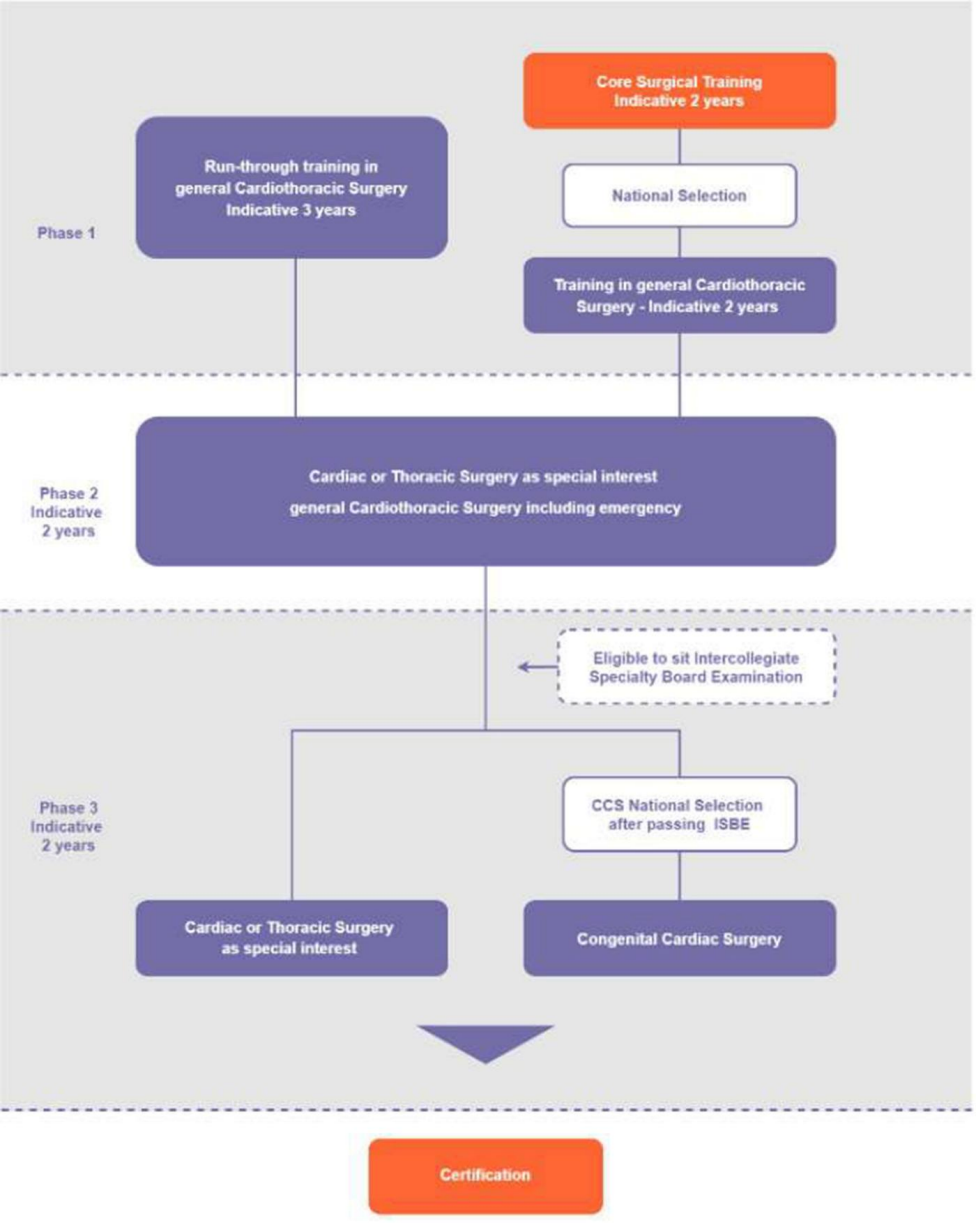
The Cardiothoracic Surgery Training Pathway. Final year of phase 1 (year 3), phase 2, and phase 3 is equivalent to the previous known higher speciality training

The CTS new curriculum was introduced after approval from the UK GMC and the MCI in 2021 to all surgical specialities.^7^ Since then, CTS has become a 7-year training programme without requiring a 2-year basic surgical training in advance. Trainees who complete the foundation programme can apply directly to the CTS training programme. The CTS training pathway is shown in **Figure 5**.

Another major change is that the curriculum transitioned from a time-based to an outcome-based model, with the expected training period reduced from 8 to 7 years. The GMC (the regulatory body) proposed shortening the length of training in all specialities, including CTS. The GMC (the regulatory body) proposed shortening the length of training in all specialities, including CTS. Although many have claimed this would increase the number of surgeons in a shorter period, this was never stated to deal with the shortfall of personnel in the United Kingdom. To meet the revised curriculum requirements, trainees must select cardiac or thoracic surgery as their special interest by their ST4. By the end of the training, trainees are expected to perform 250 major cases as first operator, with the majority in the speciality of interest (cardiac/thoracic surgery). This is similar to the recommendation by the European Association for CTS Residents Committee.^8^ The introduction of 250 index major cardiothoracic surgical cases was not included in the previous curriculum, where individual deaneries (regions) would have their standard level to determine trainees’ operative capability. A panel of surgeons assessed individual trainees annually. The review was based on trainees’ experience and a detailed report provided by their clinical supervisor.

One of the major concerns of shortening the training was whether trainees would have enough operative experience by the time they graduate. Our data demonstrate that trainees can undertake 250 major cases as the primary operator with approximately 6-7 years of higher surgical training. This raises the question of whether trainees can complete the training within the 7 years recommended by the new curriculum. The run-through training is now structured into 3 phases (**Figure 5**), with phase 1 being 3 years, replacing the previous basic surgical training of 2 years. It is intended that the phase 1 training will eliminate/minimize the need for general surgery rotation, and trainees will focus on CTS training from day 1. However, the structure of phase 1 training varies from deanery to deanery, with some using the previous basic surgical training model/rotation. Moreover, one should not forget that competence is beyond the ability just to perform “standard” procedures. Decision-making, management of complications, communication skills, and teamwork are some of the criteria defining a surgeon. Whether shortening the training duration in the new curriculum has an impact on the non-technical skills aspect remains unknown.

There has been a small number of articles discussing the operative experience for CTS trainees worldwide. Using the Accreditation Council for Graduate Medical Education case logs in the United States, Shah and colleagues reported the operative experience for traditional CTS residents in the United States, with an increase in residents, particularly thoracic-themed trainees.^9^ A survey of CTS residents in the United States reported CABG and AVR were routinely performed by graduating residents as the operative surgeons, while advanced cardiac surgical procedures were less likely to be performed by residents.^10^ A similar trend was noted with our results, where 85% of cases logged by cardiac-themed trainees were CABG and AVR. The Society for Cardiothoracic Surgery of Great Britain and Ireland has introduced several fellowships (robotic thoracic surgery, Barts/Liverpool major aortic and Bristol minimally invasive and complex mitral valve repair surgery) accredited by the Royal College of Surgeons of Edinburgh to undertake further training and prepare recent graduates to become independent consultant cardiac surgeons.^11^

The impact of COVID-19 on cardiac surgery services remains, even though the pandemic ended 2 years ago. While there is an expansion of thoracic surgical service in the United Kingdom, partly due to national lung cancer screening, the cardiac surgical volume has not yet returned to the pre-pandemic level.^12^ Abdel Shafi *et al.* have raised concerns regarding the negative impact of COVID-19 on CTS training, with further implications downstream.^2^ Our data also demonstrated an increased time to completion in cardiac-themed trainees, likely to compensate for the time lost from the pandemic. This should be recognized by the training body, and trainees impacted by COVID-19 should be appropriately supported. In the post-COVID-19 era, where the cardiac surgical volume has significantly reduced, trainees may be unable to reach the required 250 cases and may require extra time in training.

### Limitation

There were several limitations in this study. First, the surgical logbook was based on trainees’ input. Trainees who are actively logging for their cases may have a significantly higher number of cases than trainees who are less likely to do that.^13^ However, documentation of surgical cases is a compulsory requirement for all surgical trainees, and a satisfactory number of cases should be included to achieve an ARCP outcome 6 and the award of CCT. Moreover, being a competent surgeon is more than achieving surgical competency/250 major cases. The curriculum also includes requirements for professional skills, professional knowledge, capabilities in leadership and teamwork, patient safety and quality improvement, education and training, as well as research and scholarship. Surgical competency is undoubtedly an important element for trainees to be independent practitioners, but it should not be the only criterion. However, while case volume alone does not equate to surgical competency, these data can serve as a benchmark for other local and national training programs. The cardiac surgical activities have significantly reduced after the COVID-19 pandemic. Therefore, the data presented may not be applicable in the post-COVID-19 era.

## CONCLUSION

The United Kingdom and Ireland CTS surgical trainees have good exposure regarding the volume of major cases as the first operator, regardless of subspeciality choice. This supports the feasibility of gaining surgical competency in order to be awarded the CCT required in the new 2021 curriculum. However, post-COVID-19 reductions in surgical volume and the removal of basic surgical training may require integration and reform of the training programme to ensure trainees can complete the programme within the intended 7 years.

## Supplementary Material

ezaf228_Supplementary_Data

## Data Availability

The data underlying this article will be shared on reasonable request to the corresponding author.
