# Supplementary material for: An Analysis of Trainees’ Operative Experiences Over the Past 16 Years Based on the Requirements of the New 2021 Cardiothoracic Surgery Curriculum in the United Kingdom and Ireland
Source: Eur J Cardiothorac Surg. 2025 Jul 9;67(7):ezaf228. doi: 10.1093/ejcts/ezaf228 (PMC12288950; doi:10.1093/ejcts/ezaf228)
Supplement: ezaf228_Supplementary_Data [file ezaf228_supplementary_data.docx]

| **Cardiac Surgery Major Cases** |
| --- |
| ● Coronary Artery Bypass Grafting (CABG), either alone or in combination with another procedure, such as valve repair or replacement  ● Valve repair or replacement, either alone or in combination with CABG or any other cardiac procedure  ● Thoracic aortic surgery  ● Other major cardiac surgical cases involving cardiopulmonary bypass (CPB), such as post infarct ventricular septal defect (VSD) repair, excision of atrial myxoma or pericardiectomy.  ● Implantation of the heart or lung (transplantation)  ● Heart-lung block retrieval  ● Any congenital cardiac procedure (atrial septal defect closure, VSD closure, patent ductus arteriosus (PDA) ligation etc.) |
| **Thoracic Surgery Major Cases** |
| ● Anatomical lung resection (open, video-assisted thoracoscopic surgery (VATS) or robotic)  ● Correction of pectus deformity  ● Decortication  ● Thoracotomy for trauma  ● Chest wall resection and reconstruction  ● Tracheal resection  ● Surgery of secondary pneumothorax (VATS/open) |

Table 1 showing the examples of major cases listed in the 2021 Cardiothoracic Surgery curriculum.

| Outcome 1: Achieving progress and the development of competences at the expected rate |
| --- |
| Outcome 2: Development of specific competences required – additional training time not required |
| Outcome 3: Inadequate progress by the trainee – additional training time required |
| Outcome 4: Released from training programme with or without specified competences |
| Outcome 5: Incomplete evidence presented – additional training time may be required |
| Outcome 6: Gained all the required competencies for completion of training |
| Outcome 8: Out of programme (for those currently undertaking research or experience) |
| Outcome 10.1: Achieving progress, acquisition of some capabilities have been delayed by Covid, remaining training still available. |
| Outcome 10.2: Achieving progress, acquisition of some capabilities have been delayed by Covid, e.g. exams, has reached CCT date or other transition point, so additional training time is needed. |

Table 2 showing the potential ARCP outcomes awarded to trainees by the ARCP panel members
